# Precision targeting of gut-kidney axis: mucosal immunomodulation in IgA nephropathy—a perspective

**DOI:** 10.3389/fmed.2026.1784801

**Published:** 2026-03-06

**Authors:** Li-hua Sun, Xia-xia Yang

**Affiliations:** Department of Nephrology, Yan’an People’s Hospital, Yan’an, China

**Keywords:** galactose-deficient IgA1, gut-kidney axis, IgA nephropathy, immunomodulation, intestinal barrier, microbiota, mucosal immunity, targeted therapy

## Abstract

IgA nephropathy (IgAN) is a primary glomerular disease closely linked to mucosal immune dysregulation, involving a complex interplay between intestinal immune homeostasis and renal injury. The “gut-kidney axis” has recently emerged as a central conceptual framework for understanding IgAN pathogenesis, connecting intestinal mucosal immunity with kidney pathology. This perspective article scrutinizes the mechanistic role of this axis, focusing on key processes such as the production of galactose-deficient IgA1, intestinal barrier dysfunction, and gut microbiota dysbiosis. Building on this foundation, we propose “precision targeting of the gut-kidney axis” as a novel therapeutic paradigm for mucosal immunomodulation in IgAN, and discuss integrated strategies ranging from microbial and barrier modulation to specific immune interventions. By emphasizing multi-target, cross-organ approaches, this paradigm offers a promising alternative to conventional therapies and provides a translational roadmap for developing mechanism-based treatments in IgAN.

## 1 Introduction

IgA nephropathy (IgAN) is recognized globally as the most prevalent primary glomerulonephritis and represents a leading cause of end-stage renal disease (1). The disease is pathologically characterized by the deposition of immune complexes, predominantly composed of aberrantly glycosylated immunoglobulin A1 (IgA1), in the glomerular mesangium (2, 3). This deposition initiates a cascade of mesangial hypercellularity, expansion of extracellular matrix, and progressive renal impairment (2, 4). Clinically, IgAN manifests heterogeneously, ranging from isolated microscopic hematuria to rapidly progressive renal failure, contributing to its variable prognosis (5).

While historically associated with mucosal infections, particularly of the upper respiratory or gastrointestinal tract, contemporary understanding of IgAN pathogenesis increasingly centers on the “gut-kidney axis” (6–8). This paradigm highlights a functional interconnection between intestinal homeostasis and renal disease progression (8, 9). Key mechanistic insights implicate intestinal dysbiosis, compromised mucosal barrier function, and the subsequent systemic dissemination of microbial or dietary antigens in driving the production of galactose-deficient IgA1 and the formation of nephritogenic immune complexes (10).

Standard supportive care, primarily involving renin-angiotensin system inhibition, offers limited efficacy and does not target underlying immunopathogenic mechanisms (11). Although systemic immunosuppression can reduce proteinuria, its utility is constrained by significant off-target adverse effects and lack of specificity for gut-derived drivers of disease (12). The recent development of a targeted-release budesonide formulation, designed to act locally in the distal ileum, represents a mechanistically informed advance (13). However, its focus on isolated mucosal immune modulation may not fully address the multifactorial nature of the gut-kidney axis, including microbial ecology and barrier integrity (14).

**Figure 1:**
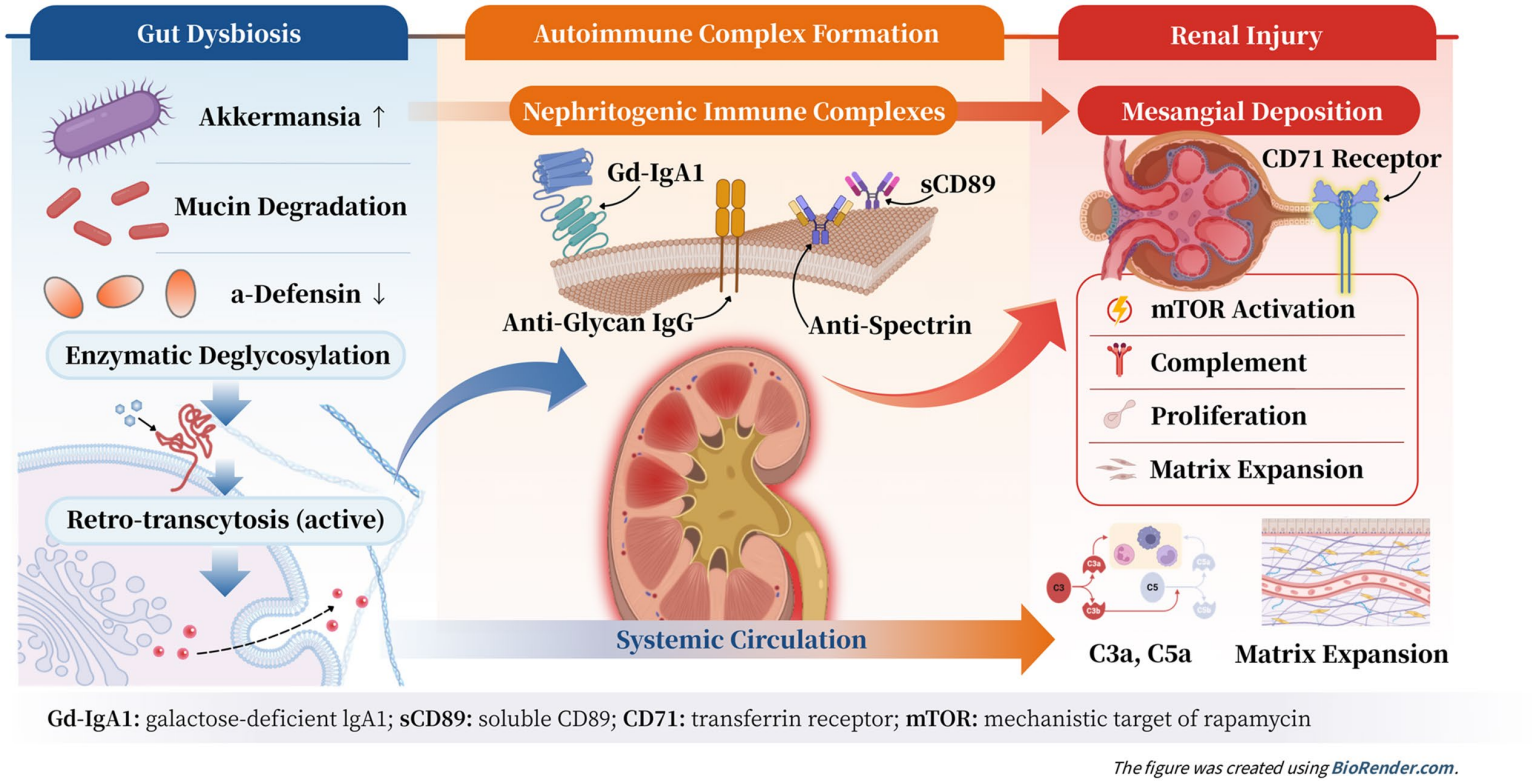
Mechanistic cascade of mucosal initiation, autoimmune amplification, and CD71-mediated renal injury in IgA nephropathy.

This perspective article aims to appraise and integrate evidence supporting “precision targeting of the gut-kidney axis” as a novel therapeutic framework for IgAN (Figure 1). We first delineate the scientific foundations linking intestinal mucosal immunity to renal injury. We then evaluate emerging therapeutic strategies within this axis, encompassing microbiota modulation, barrier restoration, and specific interference with pathogenic IgA1 synthesis or clearance. Finally, we discuss translational challenges and future directions to advance this paradigm toward clinically effective, mechanism-based therapies for IgAN.

## 2 Central mechanistic role of the gut-kidney axis in the pathogenesis of IgAN

### 2.1 Intestinal mucosal immune system: primary site of IgA production

The intestinal tract is a central hub for immune activity, housing the gut-associated lymphoid tissue where B cells mature into IgA-secreting plasma cells upon antigen exposure (15). These plasma cells predominantly produce polymeric IgA1, which is transported across the intestinal epithelium via the polymeric immunoglobulin receptor to provide frontline defense at the mucosal surface (16). In IgAN, dysregulation of this finely tuned system leads to the aberrant generation of pathogenic IgA1 (8).

### 2.2 Production and systemic trafficking of pathogenic IgA1

Generation of galactose-deficient IgA1 (Gd-IgA1): The formation of Gd-IgA1, a hallmark of IgAN, results from disrupted O-glycosylation of IgA1 molecules (17). This process is influenced by gut-derived signals, including microbial metabolites such as short-chain fatty acids and cytokines like BAFF and APRIL, which alter glycosyltransferase activity in mucosal B cells (18–20).

Mucosa-bone marrow axis: Antigen-primed lymphocytes from the intestinal mucosa can migrate systemically and home to the bone marrow, establishing a long-term reservoir of plasma cell precursors (21, 22). These cells persistently produce Gd-IgA1, sustaining its circulation even in the absence of ongoing intestinal inflammation (21).

Immune complex formation and pathogenicity: Circulating Gd-IgA1 acts as an autoantigen, leading to the formation of immune complexes with anti-glycan antibodies (21, 23). More recently, autoantibodies targeting spectrin have been detected in patients with IgAN. These antibodies may expand the variety of antigens within circulating immune complexes and exacerbate mesangial injury (24, 25). This supports the view that loss of immune tolerance in IgAN involves more than just glycan-related epitopes. Beyond antiglycan antibodies, the soluble form of the IgA Fc receptor CD89 (sCD89) has gained increasing attention as a key structural and functional component of circulating immune complexes (26). sCD89 can bind to polymeric IgA1, stabilize macromolecular aggregates, and enhance their deposition in the mesangium (27). Clinical studies have confirmed the presence of sCD89-containing complexes in both adult and pediatric IgAN, while mechanistic investigations show that these complexes directly drive mesangial proliferation and inflammatory signaling (27, 28). Thus, integrating sCD89 into the multi-hit model offers a more comprehensive understanding of immune complex pathogenicity in IgAN.

Immune complex deposition and renal injury: These pathogenic immune complexes deposit in the glomerular mesangium. The underlying mechanisms of this mesangial deposition are also becoming clearer. CD71, the transferrin receptor highly expressed on mesangial cells, has been recognized as a key mediator in binding and internalizing Gd-IgA1-containing complexes (29–31). Experimental blocking of CD71 reduces mesangial accumulation and downstream activation. Notably, signaling via this receptor intersects with the mTOR pathway, and pharmacological inhibition of mTOR has demonstrated encouraging effects in attenuating mesangial responses (32). These advances underscore receptor-targeted strategies as potential complements to upstream mucosal-based interventions. Once deposited, the complexes trigger complement activation, induce sustained mesangial cell activation and proliferation, and promote inflammation and extracellular matrix overproduction, which lead to sclerosis (31, 33, 34). This pathogenic cascade culminates in the clinical manifestations of IgAN, primarily hematuria and proteinuria (21, 35).

### 2.3 Intestinal barrier dysfunction and microbiota dysbiosis

Loss of intestinal barrier integrity, often termed “leaky gut,” is observed in subsets of IgAN patients (9). Increased intestinal permeability permits translocation of microbial antigens into circulation, provoking systemic immune activation and amplifying Gd-IgA1 production (14). Recent landmark studies have significantly advanced our understanding of how gut dysbiosis contributes to the initiation of autoimmunity in IgAN (36, 37). Independent cohorts in Europe and Asia have consistently observed an expansion of mucin-degrading bacteria—notably *Akkermansia muciniphila*—in affected patients (36, 37). Experimental evidence suggests this microbial shift is linked to impaired *α*-defensin–mediated control of bacterial growth, a defect further supported by susceptibility loci identified through genome-wide association studies (36, 38, 39).

Mechanistically, Gleeson and colleagues demonstrated that IgA1 can undergo enzymatic deglycosylation directly in the intestinal lumen, rather than solely during B-cell synthesis (36). The deglycosylated IgA1 is then actively transported across the epithelium via retro-transcytosis into systemic circulation, where it acts as a potent autoantigen that drives IgG autoantibody formation (36). It is essential to differentiate this mechanism from mere changes in paracellular permeability. Unlike the broader “leaky gut” hypothesis, experimental models indicate that the epithelial transport of deglycosylated IgA1 specifically occurs via regulated retro-transcytotic pathways (36). This highlights a process of active immunological trafficking rather than passive diffusion. These findings revise the traditional view of passive barrier leakage and instead emphasize a coordinated mucosal process that connects dysbiosis to systemic autoimmunity.

Concurrent alterations in gut microbiota composition—such as expansion of *Prevotella* species—may further stimulate pathogenic mucosal immune responses (40). The convergence of these specific bacterial taxa likely reflects a broader ecological imbalance that drives antigenic stimulation. Additionally, gut-derived uremic toxins (e.g., indoxyl sulfate) exacerbate renal damage through direct nephrotoxicity and by further compromising intestinal barrier function, establishing a vicious cycle that accelerates disease progression (36, 41). Together, intestinal barrier defects and dysbiosis form upstream drivers within the gut-kidney axis in IgAN.

Collectively, these advances support a revised understanding of IgAN as arising from a coordinated sequence of events rather than isolated abnormalities. The process begins with dysbiosis-driven enzymatic modification of IgA1 in the intestinal lumen, accompanied by impaired innate antimicrobial regulation and active epithelial retro-transcytosis, together establishing a mucosal origin for autoantigen generation. Subsequent diversification of immune recognition—through antiglycan responses, incorporation of sCD89, and additional autoantibody systems such as anti-spectrin—enhances the formation, stability, and nephritogenic potential of circulating immune complexes. These complexes are then preferentially deposited in the mesangium, a process mediated in part by CD71 and amplified through mTOR-linked intracellular signaling. This completes a continuous pathogenic axis extending from the gut to the kidney. This integrated cascade offers a contemporary and mechanistically coherent framework to guide future therapeutic innovation as illustrated in Figure 1.

## 3 Potential strategies for precisely targeting the gut-kidney axis: from mechanism to intervention

Building on the established mechanistic role of the gut-kidney axis in IgAN pathogenesis, precision interventions targeting this axis present a promising therapeutic frontier. These approaches aim to modulate upstream drivers of disease while limiting systemic immunosuppressive effects.

### 3.1 Modulating the gut microbiota

The gut microbiota plays a fundamental role in maintaining mucosal immune homeostasis. Targeted strategies for its modulation include the administration of defined probiotics, prebiotics, or synbiotics—such as butyrate-producing strains (e.g., *Clostridium butyricum*)—to reinforce the intestinal barrier, dampen inflammation, and regulate local B-cell activity (42, 43). Dietary interventions offer a practical approach; while low-protein diets may reduce exposure to immunogenic dietary antigens, high-fiber diets promote a microbiota profile associated with anti-inflammatory short-chain fatty acid production, indirectly influencing IgA synthesis and glycosylation (44, 45). For refractory cases, fecal microbiota transplantation may offer a means to broadly reset microbial communities and correct immune dysregulation, though its application in IgAN requires further clinical investigation (42, 46).

### 3.2 Restoring intestinal barrier integrity

Strengthening the compromised intestinal barrier is a direct strategy to limit antigen translocation (47). This can involve supplementation with tight junction modulators such as zinc or glutamine to enhance epithelial cohesion (48). More advanced approaches focus on the targeted delivery of mucosal protective agents—for example, using engineered formulations to deliver mucins or defensins directly to the intestinal epithelium, thereby restoring its structural and functional integrity (49).

### 3.3 Precision immunomodulation of mucosal immunity

A key objective is to achieve localized immunomodulation within the gut while minimizing systemic effects. This can be pursued through gut-restricted drug delivery systems that release low-dose immunomodulators specifically in the intestinal lumen (50). Another strategy involves targeting the BAFF/APRIL pathway locally to inhibit the differentiation and survival of Gd-IgA1-producing plasma cells (19). Furthermore, inhibiting mucosal addressing cell adhesion molecule-1 may prevent the homing of gut-primed lymphocytes to distant sites such as the bone marrow, thereby disrupting the chronic cycle of pathogenic IgA1 production (51).

### 3.4 Clearing pathogenic immune complexes

Therapeutic efforts may also focus on eliminating already formed pathogenic factors. Gut-localized B-cell or plasma cell-targeted therapies could selectively deplete the cellular sources of Gd-IgA1 (19, 52). Additionally, complement inhibitors that block the alternative pathway (e.g., anti-C5 agents) may mitigate both the renal damage from deposited immune complexes and the downstream inflammatory cascade within the gut-kidney axis (53).

Together, these targeted strategies—spanning microbial modulation, barrier restoration, precise immunomodulation, and pathogenic clearance—form a cohesive, multi-level framework for intervening in the gut-kidney axis, offering a pathway toward more effective and safer treatments for IgAN (Table 1).

**Table 1:** Precision intervention strategies targeting different levels of the gut–kidney axis in IgA nephropathy.

Target level	Pathophysiological driver	Representative strategies	Key mechanisms	Translational status
Gut microbiota	Dysbiosis, reduced SCFAs	Probiotics, diet, FMT	Restore immune tolerance	Preclinical/ early clinical
Intestinal barrier	Increased permeability	Zinc, glutamine, mucosal agents	Reduce antigen translocation	Experimental
Mucosal immunity	BAFF/APRIL overactivation	Gut-restricted immunomodulators	Reduce Gd-IgA1 production	Clinical trials
Systemic injury	Immune complex deposition	Complement inhibitors	Attenuate renal inflammation	Phase 2–3

Gd-IgA1, galactose-deficient immunoglobulin A1; BAFF, B-cell activating factor; APRIL, a proliferation-inducing ligand; SCFAs, short-chain fatty acids; FMT, fecal microbiota transplantation.

## 4 Challenges and future directions

While evidence supporting the gut-kidney axis in IgAN pathogenesis continues to accumulate, translating this knowledge into effective therapies presents distinct challenges. These obstacles simultaneously outline critical priorities for future research.

### 4.1 Scientific challenges

Significant spatiotemporal heterogeneity characterizes the gut-kidney axis, with mucosal-systemic immune interactions varying across disease stages and intestinal segments (54). This complicates the selection of optimal intervention timing and anatomical targeting. Furthermore, pronounced inter-individual differences in gut microbiota, genetic background, and environmental exposures demand personalized therapeutic approaches rather than uniform interventions (54, 55). A major translational gap remains the lack of validated, noninvasive biomarkers to assess intestinal immune activity, predict progression, or monitor treatment response objectively (56, 57).

### 4.2 Technical challenges

Pharmaceutically, achieving precise, sustained drug delivery to specific gut regions—particularly immune-rich sites like the ileal Peyer’s patches—requires advanced targeting technologies beyond current enteric-release systems (58, 59). Equally limiting are existing preclinical models; substantial interspecies differences in intestinal immunity and microbiota compromise the predictive value of animal studies for human therapeutic outcomes (60, 61).

### 4.3 Directions for clinical translation

Future translation should prioritize: (1) Developing multi-omics-based stratification integrating metagenomic, metabolomic, and immunologic profiles to guide personalized treatment (62, 63); (2) Establishing novel gut immune monitoring tools, such as circulating gut-derived IgA clonal signatures or barrier integrity markers, for noninvasive tracking (64); (3) Designing early-phase trials with mucosal endpoints (e.g., reduction in gut-derived Gd-IgA1) to accelerate mechanistic drug development (65, 66); and (4) Exploring rational combination strategies, for instance, coupling microbiota-directed therapies with localized immunomodulators, to synergistically target multiple nodes of the axis while minimizing systemic effects (62, 64, 65).

## 5 Summary

The gut-kidney axis has emerged as a pivotal conceptual framework for understanding IgAN pathogenesis and for guiding novel therapeutic development. A “precision targeting” approach to this axis constitutes a transformative therapeutic paradigm, focusing intervention upstream at the origins of disease—namely, dysregulated mucosal immunity and disrupted intestinal homeostasis. This strategy promises greater tissue specificity than conventional systemic immunosuppression, with the potential to improve efficacy while minimizing off-target effects. Translating this paradigm into clinical practice will require sustained interdisciplinary collaboration across nephrology, gastroenterology, immunology, microbiology, and bioengineering. While significant challenges remain in mechanistic understanding, patient stratification, and delivery technologies, the precise modulation of the gut-kidney axis offers a compelling pathway toward disease-modifying therapies for IgAN.

## Data Availability

The original contributions presented in the study are included in the article/supplementary material, further inquiries can be directed to the corresponding author.
